# fNIRS Monitoring of Infant Prefrontal Cortex During Crawling and an Executive Functioning Task

**DOI:** 10.3389/fnbeh.2021.675366

**Published:** 2021-08-13

**Authors:** Hannah Weibley, Mina Di Filippo, Xinran Liu, Lillian Lazenby, Jackson Goscha, Alyssa Ferreira, Laura Muscalu, Nancy Rader

**Affiliations:** Psychology Department, Ithaca College, Ithaca, NY, United States

**Keywords:** fNIRS, executive function, prefrontal cortex, locomotion, attention, infancy

## Abstract

Functional near-infrared spectroscopy (fNIRS)is a brain-imaging technology used to reveal brain activity by measuring blood oxygenation. Using fNIRS we measured activity in the left prefrontal lobe of 8–14 month-old infants as they crawled or were pushed in a stroller and as they were given a passive attention task or an active executive function (EF) task. For each task, we measured peak total hemoglobin concentration and peak Oxy relative to baseline. Results revealed differences in peak Oxy levels for crawling vs. strolling and between the EF and passive cognitive tasks, with total hemoglobin greater for the EF task than the passive attention task. These results support the theoretical view that both active locomotion and EF engage the prefrontal cortex (PFC) during early development.

## Introduction

In both children and adults, the prefrontal cortex (PFC) is an area linked to executive functioning, a set of neurocognitive and regulatory processes. There are three main components to executive functions (EF): inhibitory control, cognitive flexibility, and working memory. Each function plays a role during situations that require controlled processing and selective attention. Executive functions allow us to self-regulate, task switch, problem-solve, and are overall essential to learning. They can also serve as a predictor in childhood for later cognitive development and quality of life (Diamond, 2013). However, very little is known about the development of executive function in infancy.

Koziol et al. (2011) have proposed that executive function processes in the human brain developed over evolution through pressures derived from the need to control motor behavior. This perspective links self-guided locomotion and the development of the executive function, with both functions making use of the prefrontal lobe of the brain (e.g., Koziol and Lutz, 2013). While Koziol et al. (2011) claim that locomotion and executive function share brain areas, there is no research that directly evaluates their claim for this sharing in human infants. Indirect support for this view was found in research with non-crawling infants who began at 5 months of age using a robotic locomotive device. After 2 months infants who locomoted with the robotic device performed better on executive function tasks at 7 months compared with infants who did not have that locomotive experience (Rader et al., 2019). Given the relationship reported between motor impairments and delays with poor executive function (Leonard, 2016), Koziol et al.’s (2011) theory has important clinical implications as well as serving as a potential key to understanding the development of executive function in a typically developing population. The key aim of the reported research was to explore, using functional near-infrared spectroscopy (fNIRS), the prefrontal lobe as a shared brain area for intentional locomotion and executive function in infants.

The cortical activity accompanying executive functions can be measured with technology such as fMRI and fNIRS. These technologies provide data that reflect the brain’s responsiveness to stimuli or processes needed for performing particular actions. fNIRS technology has become an increasingly popular neuroimaging technique, particularly within pediatric populations (Aslin, 2012), as it is safe and allows for subject movement. León-Carrión and León-Domínguez (2012) verified fNIRS as an accepted neuroimaging technique that could be applied in both clinical and research settings.

When neurons are activated, their metabolic demands change, resulting in an increase in oxygen consumption, local cerebral blood flow, and oxygen delivery (Lloyd-Fox et al., 2010). According to Villringer and Chance (1997), a typical hemodynamic response to neurons being activated in the prefrontal cortex (PFC) is an increase in blood flow, generating an increase in oxygenated hemoglobin (oxy-Hb), as well as a slight decrease in deoxygenated hemoglobin (deoxy-Hb) and an overall increase in total hemoglobin (HbT). In research with adults, it has been established that fNIRS can be used to measure cognitive workload (Ayaz et al., 2012) and task difficulty (Izzetoglu et al., 2004) by measuring changes in oxy-Hb, deoxy-Hb, and HbT in the prefrontal cortex (Herff et al., 2014). Also, it has been demonstrated that increased cognitive demands result in increased activation of Brodmann Area 10 (BA10), located in the prefrontal cortex (Arsalidou et al., 2013). Wager et al. (2004) associate BA10 with executive functioning and Wager et al. (2005) describe BA10, specifically the left anterior PFC, as active during tasks that require the inhibition of learned rules/behavior.

Researchers have also used fNIRS to measure activation in the prefrontal cortex during motor activities in adults. For example, Suzuki et al. (2004) investigated prefrontal cortex activation while participants walked and ran on a treadmill and found that the prefrontal cortex, as well as the premotor cortex, showed activation when adjusting to accelerating speeds on the treadmill. Lloyd-Fox et al. (2008) recorded prefrontal activity during external perturbation trials, which revealed that the prefrontal cortex plays an important role in balance control. Using a clinical population, Maidan and colleagues (e.g., Maidan et al., 2015, 2016, 2017) have reported a connection between BA10 and locomotor control in Parkinson’s Disease patients.

Two issues arise when recording from a single brain area. One is that it is not possible to know the contribution of other areas of the brain. In the research reported here, the goal has been to examine the extent to which the prefrontal cortex is activated during goal-directed locomotion and executive functioning without analyzing the contribution of other brain areas. Aslin (2012) has raised a related concern; Aslin questions the extent to which any increase in brain activity in a particular region might be explained by its role as a general index of attention or arousal. Laeng et al. (2012) have presented a case for using pupil dilation to track arousal since it is a response created by norepinephrine produced by the locus coeruleus, a structure in the brainstem that regulates the integration of the brain’s attention system, stress response, and arousal regulation. Rader and Zukow-Goldring (2015) have successfully used pupil dilation with infants as a measure of arousal. That activity in the prefrontal cortex as measured using fNIRS and pupil dilation can be separated is shown by YTS Brighter and Rader (2018) who found different patterns in pupil diameter and oxygenation response in the participants’ reactions to editing shifts in video segments, suggesting differences between pupil diameter and fNIRS in terms of the psychological phenomena they measure.

In this study, we used fNIRS to record activity from the prefrontal cortex of crawling infants during active, goal-directed, locomotion, where they crawled to reach a parent, and passive locomotion, where they were pushed in a stroller to reach a parent. The prefrontal activity was also measured during two cognitive tasks, one that involved passive attention to a dancing cat puppet and the other that involved executive function. For the executive function task, known as a “switch task,” participants learned a rule and then had to inhibit it and switch to a new rule (Kovács and Mehler, 2009). Additionally, we measured pupil diameter during the cognitive tasks to provide an index of arousal.

We hypothesized that there would be higher peak Oxy (the difference between oxy-Hb and deoxy-Hb) and HbT levels relative to baseline when infants actively crawled compared to being passively moved in a stroller. We also predicted that these oxygenation measures would be higher, compared with baseline, during the Switch Task as compared with the Cat Puppet Task. Additionally, we set out to determine the relationship between the fNIRS effects and pupil diameter.

## Materials and Methods

### Participants

Inclusion criteria for this study required participants to be within the age range of 8–15 months old, typically developing, crawling at the time of participation, able to tolerate wearing the apparatus, and able to participate in both sessions of the study. Of the 20 infants who met these criteria and came to the lab, data from eight infants could not be analyzed either because of infant fussiness that occurred during testing (five infants) or because of experimenter error (three infants).

The 12 infants whose data are described here were six males and six females, aged 8.3–15.4 months, with an average age of 11.1 months (SD = 2.17). Ten infants were identified as Caucasian and two as an ethnic minority, reflective of the local population.

Parents of the participants gave informed consent prior to their child’s participation in the study. Parents received a $20 gift card following each of the two sessions, a certificate of completion, and a photo for their time and contribution to the research. Most participants were recruited through the campus online newsletter, use of local parental pages, and other social media platforms. Approval for this research was received from the Ithaca College Institutional Review Board.

### Design and Apparatus

This study used a within-subjects design to collect data on the 12 infant participants. The experiment was carried out in two separate sessions, completed no more than 2 weeks apart. Session 1 provided measures of oxygenation levels in the brain during crawling and stroller movement, while Session 2 provided measures of oxygenation levels in the brain during a passive attention task and during an active cognitive task requiring executive function.

### Functional Near-Infrared Spectroscopy

fNIRS technology uses LED light and optical sensors to measure the concentration changes from baseline in oxygenated hemoglobin (oxy-Hb) and deoxygenated hemoglobin (deoxy-HB) in the capillaries (León-Carrión and León-Domínguez, 2012). Blood oxygenation can be differentiated due to oxy-Hb and deoxy-Hb having different absorption properties to near-infrared light exposure and varying light scattering patterns when the light is reflected back from the brain (Lloyd-Fox et al., 2010).

**Figure 1:**
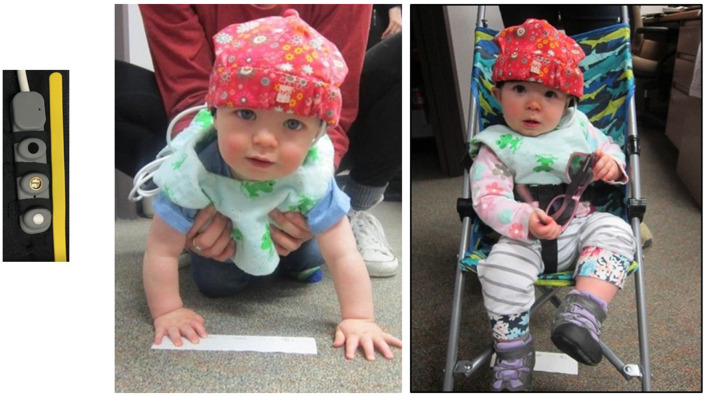
Pediatric sensor strip and infants in the crawling and stroller conditions.

Our fNIRS device (Biopac Systems, 2018) emits infrared light having wavelengths of 730 nm and 850 nm, under which biological tissue essentially becomes translucent (Lloyd-Fox et al., 2010). We used a Biopac pediatric sensor (RXFNIR-PED) to measure light levels reflected back from the oxygenated and deoxygenated hemoglobin; this sensor has two channels, two detectors, and one emitter, with an inter-optode distance of 20 mm (see Figure 1). The sensor is sized for infants to record from a single hemisphere of the prefrontal cortex.

Oxy-Hb and deoxy-Hb levels were measured within a light level of less than 4,000 mv. To operate within this range, the LED current (brightness) and detector gain (sensitivity) were adjusted for each participant as determined by CobiStudio, software distributed by Biopac (Ayaz, 2005); typical brightness (LED current) was 12 mA and a gain of 2. Because we were working with infants, a sample rate of 50 per optode per frame was used. Using CobiStudio, event markers were programmed manually to signify the beginning and end of specific tasks within the study. Markers were also used to indicate any intervals in which the infant was briefly fussing or crying, and these intervals were excluded from analyses. Infants who fussed or cried repeatedly during a trial are not included in our sample.

Each data output file was refined using a low pass filter, which was created for the pediatric wireless device, and a motion artifact rejection algorithm. The low pass filter was set at 0.1 Hz with an order of 50, Hamming. The motion artifact rejection filter [Sliding-Window Motion Artifact Rejection (SMAR)] eliminates any data that are associated with light oversaturation and other abnormalities caused by the movement of the sensor on the skin. Details of these filters and the Modified Beer Lambert Law used can be found in Ayaz (2010) and Ayaz et al. (2010). Final analyses of the infrared light and oxygenation data were conducted using fNIRSoft software.

We recorded hemodynamic responses from the left anterior prefrontal cortex. While it cannot be definitively determined for infants, we believe we measured responses from BA 10, comprising the most anterior part of the prefrontal cortex. The fNIRS pediatric sensor was placed over the left side of the infant’s forehead, centered just above the pupil. The sensor was held in place by a black headband and then covered with and a cotton hat (see Figure 1) to secure the sensor strip and to prevent light leakage. The sensor information was transmitted to a computer *via* a wireless interface secured behind the infant’s back in the zippered pouch of a custom-made vest.

The hemodynamic measures used as dependent variables in this research were Peak Oxy and Peak HbT. A peak indicates the maximum value obtained during a particular task. The peak response was selected as a dependent measure value because the response in infants over time is unknown and variable. Using a peak measure allows a fair comparison across infants and also across tasks. Oxy levels reflect changes in oxygenation concentration determined by the difference between oxy-Hb and deoxy-Hb relative to baseline. HbT levels indicate the total hemoglobin concentration, relative to baseline, and reflect changes in blood volume. The final 3 s of baseline was used in all cases for the data analyses.

### Procedure

#### Session 1

**Figure 2:**
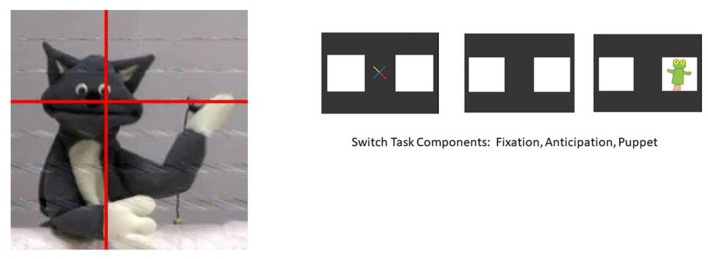
The cat puppet and switch task.

The focus of the study’s first session was locomotion. After the fNIRS device was placed on the participant’s forehead with the emitter placed directly over the pupil of the left eye, they were brought into the testing room for the locomotion trials. For the passive movement portion, the participant was pushed in a stroller for a distance of 12 feet towards their parent who called to them and/or enticed them with a toy (see Figure 2). For the active movement portion, the participant crawled 12 feet towards their parent who encouraged them in a fashion similar to the stroller condition. The fNIRS baselines lasted 15 s and were established during the time that the participant was stationary prior to locomotion. The order of crawling and stroller events was counterbalanced across participants. We allowed for two trials each of crawling and strolling with the intention of having one trial without recording issues.

#### Session 2

The focus of the second session was on responses to cognitive tasks. Participants were placed in a car seat in a testing room with a large plasma screen on which video segments were shown. An eyetracking system (Applied Science Laboratory) was used to track infant eye movements in relation to the video scenes so that it could be determined in real time that the infant was paying attention to the video being presented. A brief clip from a show called *Teletubbies* was shown initially to obtain the participant’s attention, establish a baseline of oxygenation levels in the prefrontal cortex, and allow experimenters to capture the participant’s eye for eyetracking.

Following *Teletubbies*, two different cognitive-visual tasks occurred. The first task, the Cat Puppet Task, displayed a cat puppet that danced to music for 15 s. This task reflects passive attention to a stimulus. The second task, the Switch Task, reflects active attention, requiring infants to learn a rule and then inhibit the learned rule and switch to a new rule. Kovács and Mehler (2009) present this task as a type of executive function task. At the start of the task, a puppet appears on the right side of the screen; this occurs for nine consecutive trials (Pre-Switch). After the ninth trial, the puppet switches to the left side of the screen, appearing for another nine consecutive trials (Post-Switch). At the beginning of each trial, a visual cue appears in the center of the screen to capture the attention of the infant and bring the focus back to the center of the screen. The total duration for each Switch Task component was 54 s (see images for both Tasks in Figure 2).

## Results

**Figure 3:**
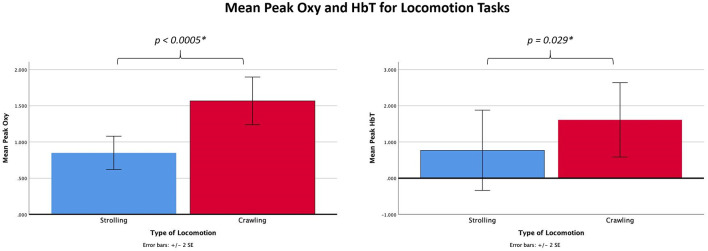
Graphs showing the mean peak Oxy and HbT for the locomotion tasks.

Comparisons between crawling and stroller locomotion were performed on data from 11 of the 12 participants, as one infant had difficulty completing the crawling task. Two paired-samples *t*-tests were conducted to compare peak Oxy and peak HbT levels (the dependent varriables) between crawling and stroller trials (levels of the independent variable). Significant differences between the stroller and crawling tasks were found for both peak Oxy, *t*_(10)_ = 6.85, *p* < 0.005, *η*^2^ = 0.824, and peak HbT, *t*_(10)_ 2.55, *p* = 0.029, *η*^2^ = 0.395. For peak Oxy, means (standard deviations) were 0.85 (0.38) for strolling and 1.57 (0.55) for crawling. For peak HbT means (standard deviations) were 0.77 (1.84) for strolling and 1.61 (1.70) for crawling. These results are shown in Figure 3.

**Figure 4:**
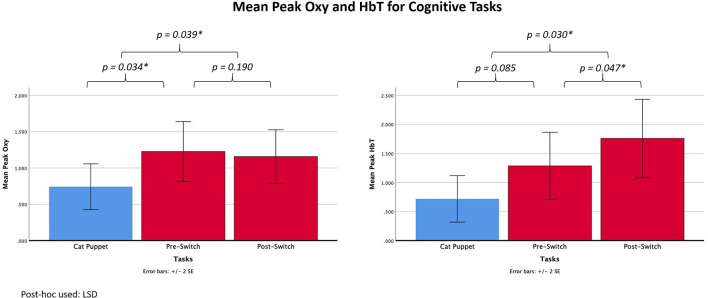
Graphs showing mean peak Oxy and HbT for the cognitive tasks.

Two repeated-measures ANOVAs with one independent variable (Task) were performed to analyze peak Oxy and peak HbT (the dependent variables) during the three cognitive tasks (the Cat Puppet, Pre-Switch, and Post-Switch). There was an overall Task effect for Oxy, *F*_(2,22)_ = 3.836, *p* = 0.037, *η*^2^ = 0.259. Subsequent paired comparisons using LSD showed that the values for the Cat Puppet Task differed significantly from the Pre-Switch (*p* = 0.034) and Post-Switch (*p* = 0.039) Tasks; the Pre-Switch Task did not differ significantly from Post-Switch (*p* = 0.717). Means (standard deviations) were 0.7427 (0.5451) for the Cat Puppet Task, 1.228 (0.7126) for Pre-Switch, and 1.1572 (0.6403) for Post-Switch, as shown in Figure 4. There was also an overall task effect for HbT, *F*_(2,22)_ = 5.282, *p* = 0.013, *η*^2^ = 0.324. Paired comparisons using LSD showed that the Cat Puppet Task differed significantly from Post-Switch (*p* = 0.039) but did not reach a value of *p* < 0.05 in comparison with Pre-Switch (*p* = 0.085). Post-Switch values differed significantly from Pre-Switch (*p* = 0.047) values. Means (standard deviations) were 0.7189 (0.6938) for the Cat Puppet Task, 1.2871 (1.000) for Pre-Switch, and 1.759 (1.1663) for Post-Switch, as shown in Figure 4.

We performed correlation analyses to look at the relationship of pupil diameter to the hemoglobin measures during the cognitive tasks. The pupil diameter values were provided by Gazetracker software by Eye Response Technologies and acquired as part of Applied Science Laboratory’s eye tracking system. No statistically significant correlations were found between average pupil diameter and peak Oxy or between pupil diameter and peak HbT. For the Cat Puppet Task, the *r*-value for Oxy was 0.26 and for HbT 0.22; for Pre-Switch the *r*-values were 0.56 and 0.55, while for Post-Switch they were 0.19 and 0.31. Of interest is the fact that the pupil diameter values for the Cat Puppet Task and both Switch Tasks were significantly correlated—Cat Puppet with Pre-Switch (*r* = 0.79, *p* < 0.01) and with Post-Switch (*r* = 0.61, *p* < 0.05) and Pre-Switch with Post-Switch (*r* = 0.77, *p* < 0.01).

## Discussion

The goal of this study was to examine the link of the prefrontal cortex to both self-guided, goal-directed locomotion and executive functioning during infancy. The data support our hypothesis that there would be higher Oxy levels when infants actively crawled compared to when they were passively moved in a stroller. Additionally, the data support the prediction of higher Oxy levels during the Switch Task as compared with the Cat Puppet Task. Moreover, based on our data, the differences in peak HbT levels between the Cat Puppet Task and the Switch Task suggest that more cognitively demanding tasks enlist a higher blood volume.

The findings of our study support Koziol and Lutz’s (2013) hypothesis that both active locomotion and executive function tasks engage the prefrontal cortical area of the infant brain. In a goal-oriented activity like crawling to a parent, one cannot separate the effect of the motor activity from the controlling of that activity. According to Koziol and Lutz’s (2013) theory, the need to control body movements for effective locomotion in infancy has recruited a brain area within the prefrontal cortex, namely BA 10, that has subsequently become the locus of more general executive function control. Indeed, our results show that an activity that requires mental coordination and inhibition of an established behavior (the Switch Task) and an activity that required motor coordination and control (goal-directed crawling) activate more strongly an area of the prefrontal cortex than passive activities (being pushed in a stroller or viewing the Cat Puppet dancing). This finding has implications for understanding the reported developmental relationship between delayed or impaired motor function and cognitive skills (Leonard, 2016).

It could be argued that in the present study the hemodynamic patterns observed during crawling and during the Switch Task reflect a higher level of attention/arousal rather than executive control, unnecessary during the Cat Puppet Task. However, the high correlations for pupil diameter across the three cognitive tasks suggest that the differences across these tasks in the hemodynamic responses cannot be completely due to differences in arousal/attention.

While not uncommon in fNIRS research, a limitation of this study is its small sample size and limited representation of ethnic groups. Also, we measured activity only in the left hemisphere because we found during pilot testing that infants were unable to tolerate the doubling of time required to measure from both hemispheres. The need for the doubled time occurs because the pediatric sensor allows for measurements from only one hemisphere. One rationale for using the left hemisphere rather than the right hemisphere is Wager et al.’s (2005) finding with adults that the left anterior PFC is active during tasks that require the inhibition of learned rules/behavior.

With these limitations in mind, we have found, for crawling-aged infants, that the left anterior prefrontal cortex is more likely to be engaged during both active locomotion and a cognitive task requiring executive function than when passively moved through the same environment or when watching a video requiring only passive attention. Future research might be directed towards looking at the hemodynamic responses in other areas of the brain to get a more complete picture of the complexities of the development of executive function in infants.

## Data Availability Statement

The raw data supporting the conclusions of this article will be made available by the authors, without undue reservation.

## Ethics Statement

The studies involving human participants were reviewed and approved by Institutional Review Board Ithaca College. Written informed consent to participate in this study was provided by the participants’ legal guardian/next of kin. Written informed consent was obtained from the minor(s)’ legal guardian/next of kin for the publication of any potentially identifiable images or data included in this article.

## Author Contributions

Suzanne Zuckerman began this research as her senior honors project at Ithaca College, troubleshooting the fNIRS set-up, developing the testing protocol, and conducting pilot research. The first six authors (HW, MD, XL, LL, JG, and AF) participated in testing infants and in contributing to the manuscript for this article. In addition, HW and MD acquired and applied their knowledge of fNIRS principles and along with XL, coded the data and carried out the statistical analyses. MD acquired the pupil diameter data and carried out the pupil diameter analysis. LM and NR supervised all aspects of the conduction of this research. All authors contributed to the article and approved the submitted version.

## Conflict of Interest

The authors declare that the research was conducted in the absence of any commercial or financial relationships that could be construed as a potential conflict of interest.

## Publisher’s Note

All claims expressed in this article are solely those of the authors and do not necessarily represent those of their affiliated organizations, or those of the publisher, the editors and the reviewers. Any product that may be evaluated in this article, or claim that may be made by its manufacturer, is not guaranteed or endorsed by the publisher.
